# Roles of PI3K pan-inhibitors and PI3K-δ inhibitors in allergic lung inflammation: a systematic review and meta-analysis

**DOI:** 10.1038/s41598-020-64594-0

**Published:** 2020-05-06

**Authors:** Jong Seung Kim, Jae Seok Jeong, Sam Hyun Kwon, So Ri Kim, Yong Chul Lee

**Affiliations:** 1 Department of Otorhinolaryngology, Chonbuk National University Medical School, Jeonju, South Korea; 2 Department of Internal Medicine, Research Center for Pulmonary Disorders, Chonbuk National University Medical School, Jeonju, South Korea; 3 Research Institute of Clinical Medicine of Chonbuk National University-Biomedical Research Institute of Chonbuk National University Hospital, Jeonju, South Korea

**Keywords:** Chronic inflammation, Asthma, Chronic inflammation

## Abstract

Meta-analysis can be applied to study the effectiveness of the summary estimates for experimental papers, producing objective and unbiased results. We investigated the effects of phosphoinositide-3-kinase (PI3K) on the inflammatory profile in allergic mouse models, which are currently under development in signal transduction materials. PubMed, EMBASE and Web of Science databases were searched for relevant literature using the search terms “ PI3K inhibitor” and “allergy” or “asthma”. Cochrane Review Manager and R were used for handling continuous variables. The primary outcomes of the inflammatory profile were divided into cell counts and inflammatory cytokines. We used a random effects model to draw a forest plot. Through the database search and subsequent selection, 17 articles were identified. Regarding the cell counts, both the PI3K pan-inhibitors and PI3K-δ inhibitors effectively reduced the total cell counts, eosinophils, neutrophils and lymphocytes. In contrast to PI3K-δ inhibitors, PI3K pan-inhibitors effectively reduced macrophages. Regarding the inflammatory cytokines, PI3K pan-inhibitors and PI3K-δ inhibitors effectively reduced total IgE, IL-4, IL-5, IL-13, TNF-α, IL-1β, VEGF and had no effect on IL-6. Compared to the PI3K pan-inhibitors, which block all pathways, selective PI3K-δ inhibitors are expected to be relatively less toxic. Regarding the efficacy, PI3K-δ inhibitors have at least the same or better efficacy than PI3K pan-inhibitors in effector cells and inflammatory mediators.

## Introduction

Meta-analysis is a method of collecting and organizing data based on existing documents and deriving the most objective and unbiased results in a transparent way. As is well known, existing meta-analyses in Cochrane Reviews have used interventional meta-analytic methods. Interventional meta-analysis is mainly used to ensure the internal legitimacy of study results. Meta-analysis can be applied to determine the effectiveness of the summary estimates for the experimental papers included in the analysis, and thus the objective basis of the study is secured^1^. Clinical trials in the area of pathophysiology are particularly limited, while animal studies can investigate the pathophysiology more extensively. These animal studies are somewhat time consuming, difficult, less reproducible, and costly. Applying meta-analysis in animal studies dealing with pathophysiology can save costs and synthesize the results of each discrete study, resulting in very meaningful final results.

Class I phosphoinositide 3-kinases (PI3Ks), lipid signalling kinases acting adjacent to cell membrane, have emerged as a promising therapeutic target for allergic inflammatory disorders because they are implicated in a broad aspect of cellular pathophysiology, and that interfering this signalling allows the development of potent anti-inflammatory agent effective against broad spectrum of disease severity^2^. PI3Ks are divided into four subtypes (α, β, γ, δ). Whereas PI3K-α and -β isoforms are present everywhere, PI3K-δ is an isoform which is uniquely distributed in circulating haematogenous cells and endothelial cells, making it particularly attractive druggable target for immune/inflammatory disorders including bronchial asthma^3^. Since the beginning of the research, therapeutic blockade of pan-class I PI3Ks have been vastly studied in oncology such as solid cancer and lymphoma, and researches have been also conducted in the field of allergy^4^. As for PI3K-δ isoform, it is also increasingly recognized as a critical mediator of severe allergic inflammation in upper respiratory tract as well as lower airways^2,5^. However, at the same time, there are concerns on the blockade of various isoforms of PI3Ks for therapeutic purposes due to its potential to disturb protective immune and inflammatory responses against diverse pathogens. In this regard, comparative analysis of the therapeutic effects of PI3Ks pan-inhibition and PI3K-δ selective blockade on different aspects of immune/inflammatory process using validated method is important for a novel drug development for the disease.

Based on this knowledge, through a meta-analysis on the pre-existing preclinical data, we investigate the therapeutic effects of both PI3K pan-inhibitor and PI3K-δ selective inhibitor on features of allergic lung inflammation including inflammatory cell infiltrations into lungs and levels of various pro-inflammatory cytokines/chemokines in several murine models of allergic lung inflammation.

## Methods

### Search strategy

This study was the subject of IRB exemption from Chonbuk National University Hospital, and followed the PRISMA guidelines^6^. PubMed, EMBASE, Web of Science, Google Scholar, and Cochrane Library databases were included in the literature search. The search term in Medline was: (PI3K inhibitor OR idelalisib) AND (asthma OR allergic OR allergy). All the PI3K pan-inhibitor and PI3K-δ selective inhibitor found in our search criteria. Similar search terms were used for the four other databases. Two authors independently performed the literature search and the published studies identified in the search results included data up to July 2019.

### Selection of individual studies

The inclusion criteria for studies were that they: (1) discussed the PI3K signalling pathway; (2) discussed allergies or asthma; (3) were in English; (4) contained meaningful data (cell counts and inflammatory cytokines); (5) included an allergy or asthma mouse model.

Exclusion criteria for studies were that they: (1) did not mention PI3K inhibitor; (2) discussed leukaemia or other malignancies; (3) did not contain appropriate data; (4) were review articles; (5) discussed organs other than those related to asthma; (6) did not address the inflammatory profile; (7) included animal models other than allergy or asthma; or (8) dealt with PI3K-δ and γ dual inhibitor.

We divided the PI3K inhibitors into two groups as follows: (1) drugs that inhibited all PI3K isoforms (α, β, γ, and δ) were regarded as PI3K pan-inhibitors; (2) drugs that specifically inhibited PI3K-δ were termed PI3K-δ inhibitors. Individual studies were selected only when used in an allergic lung inflammatory mouse model.

### Data extraction

Three authors independently screened the study titles and abstracts (JSK, JSJ, SHK). The following information was extracted from each study: author names, year of publication, allergy model, PI3K drug name, dose used, inflammatory profile, duration of study, population used. When any disagreement occurred, the other authors acted as arbitrators.

### Statistical analysis

Cochrane Review Manager and R were used for statistical analysis. First, we found the mean and SD values for the continuous variables in the inflammatory profile in the original text. If the data were not presented numerically in the original paper, the mean and SD values were calculated from a graph using the Graph Data Extractor^7^. The primary outcomes of the inflammatory profile were as follows: total cell counts, eosinophils, neutrophils, macrophages, lymphocytes, total IgE, IL-4, IL-5, IL-13, eotaxin, IFN-γ, IL-6, TNF-α, TGF-β, IL-1β, VEGF. Data were analyzed as continuous variables. Standard mean differences (SMD with 95%CI), Cohen’s d and Hedge’s g were calculated from the mean and SD values^8,9^. Standard error (SE) was calculated by the inverse variance method. The I^2^ value was used to assess inter-study heterogeneity. We used a random effects model to draw a forest plot, which we considered to be a suitable model for animal studies where there may be many heterogeneities^1^.

### Mathmatical formula in this meta-analysis

We followed standard meta-analysis techniques^1,9,10^. The effect size from each individual independent study was obtained as outlined next.

The variables used in this study are all continuous variables, and *d* is the standardized mean difference between the two groups ${\bar{X}}_{1}$ and ${\bar{X}}_{2}$.

1 $$d=\frac{{\bar{X}}_{1}\mbox{--}{\bar{X}}_{2}}{{S}_{within}}$$

2 $${S}_{within}=\sqrt{\frac{({n}_{1}\mbox{--}1){S}_{1}^{2}+({n}_{2}\mbox{--}1){S}_{2}^{2}}{{n}_{1}+{n}_{2}\mbox{--}2}}$$

In the numerator, ${\bar{X}}_{1}$ and ${\bar{X}}_{2}$ are the sample means in the two groups. In the denominator, *S*_within_ is the within-groups standard deviation, pooled across groups, where *n*_1_ and *n*_2_ are the sample sizes in the two groups, and *S*_1_ and *S*_2_ are the standard deviations in the two groups.

Variations of *d* are as follows.

3 $${V}_{d}=\frac{{n}_{1}+{n}_{2}}{{n}_{1}{n}_{2}}+\frac{{d}^{2}}{2({n}_{1}+{n}_{2})}$$

4 $${W}_{i}^{\ast }=\frac{1}{V{Y}_{i}^{\ast }}=\frac{1}{V{Y}_{i}+{T}^{2}}$$

The total variance for a study $V{Y}_{i}^{\ast }$ is the sum of the within-study variance (*VY*_*i*_) and the between-studies variance (*T*^2^). This method of estimating the variance between studies is the most popular, and is known as the DerSimonian and Laird method^9^.

To combine the studies, we used a random-effects model in which studies are weighted by the sum of the true variation among studies and the sampling variation within studies. The effect sizes were combined across studies to give a weighted mean effect size across *K* studies:

5 $$\hat{\mu }=\frac{{\sum }_{i=1}^{K}\,{w}_{i}{\hat{\theta }}_{i}}{{\sum }_{i=1}^{K}\,{w}_{i}}$$

6 $${S}_{\hat{\mu }}^{2}=\frac{1}{{\sum }_{i=1}^{K}\,{w}_{i}}$$

where *θ* is the effect size of each gene in each study, and *w*_i_ is the corresponding weight for that study.

## Results

### Search results

Using the search terms as discussed earlier, a total of 771 articles were identified: 184 articles in Medline, 241 articles in Web of Science, 151 articles in EMBASE, 192 articles in Google Scholar, and 3 articles in Cochrane Library. From these, the title and abstract were checked, and 540 articles were excluded as irrelevant studies, leaving 231 articles. Of these, 176 articles were excluded as duplicate papers, leaving 55 full length articles. Of these, a total of 38 papers were excluded: 3 papers on leukaemia, 12 papers without data, 14 papers with insufficient data, and 4 papers with duplicate data. Thus, a total of 17 papers were left for qualitative and quantitative evaluation (Tables 1 and 2)^11–27^.

**Table 1:** General characteristics of PI3K pan-inhibitors in mouse models.

Author	Publication year	Model	Fluid or blood	N Exp	N Con	Drug	Dose	Primary outcomes
Campa^11^	2018	OVA induced C57BL/6J and BALB/c mice	BALF	10	6	CL27c	2 mg/mL	E, N, L, M, T, 5, 13
Wagh^12^	2017	OVA induced BALB/c mice	BALF	6	6	INK654	30 mg/kg	E, N, L, M, T, α, 2, 5, 6, γ
Huang^13^	2017	IL-25 induced BALB/c mice	BALF	5	5	LY294002	80 μg/50 μL	E, N, L, T, α, 5, 6, 13, Eo, 1β, β, V
Oikawa^14^	2016	OVA induced C57BL6 mice	BALF (cytokines), blood (cell counts)	6	6	ZSTK474	30 mg/kg	E, T, 4, 5, 13, β
Saw^15^	2016	Cockroach induced BALB/c mice	BALF (cell counts, cytokines), blood (I)	6	6	LY294002	3 mg/kg	E, N, T, 4, 5, 13, 10, 12, I
Yao^16^	2015	TDI induced BALB/c mice	BALF (cell counts), blood (I)	8	8	LY294002	1.5 mg/kg	E, N, L, M, T, I
Liang^17^	2015	TDI induced BALB/c mice	BALF (cell counts,4), blood (I)	7	7	LY294002	1.5 mg/kg	E, N, L, M, T, I, 4
Choi^18^	2013	OVA induced BALB/c mice	BALF	5	5	LY294002	1.5 mg/kg	E, N, L, M, T, 4, 5, 13, α, 1β, Eo
Xia^19^	2012	OVA induced rat	BALF	8	8	Wortmannin	15 μg/kg	E, N, L, M, 4, γ
Duan^20^	2005	OVA induced BALB/c mice	BALF	6	6	LY294002	3.75 mg/kg	E, N, L, M, T, 4, 5, 13, Eo, γ
Kwak^21^	2003	OVA induced BALB/c mice	BALF	6	6	Wortmannin	100 μg/kg	E, N, L, M, T, 4, 5

Abbreviations: Exp: experimental; Con: control; BALF: bronchoalveolar lavage fluid; OVA: ovalbumin; TDI: toluene diisocyanate; E: eosinophils; N: neutrophils; L: lymphocytes; M: macrophages; T: total cell counts; I: IgE; α: TNF-α; 2: IL-2; 4: IL-4; 5: IL-5; 6: IL-6; 10: IL-10; 12: IL-12; 13: IL-13; γ: IFN-γ; β: TGF-β; 1β: IL-1β; Eo: eotaxin; V, VEGF.

**Table 2:** General characteristics of PI3K-δ inhibitors in mouse models.

Author	Publication year	Model	Fluid or blood	N Exp	N Con	Drug	Dose	Primary outcomes
Lee^22^	2016	Fungus induced C57BL/6 mice	BALF, blood (I)	5	5	IC87114	0.1 mg/kg	E, N, L, T, 4, 5, 13, I
Collmann^23^	2013	p110dDA mice	BMMC, HUVEC	3	3	IC87114	—	6, α
Kang^24^	2012	Cockroach induced BALB/c mice	BALF, lung tissue	14	12	IC87114	10 μg	E, N, L, T, 4, 5, 13, Eo
Lee^25^	2006	OVA induced BALB/c mice	BALF	8	8	IC87114	0.1, 1 mg/kg	E, N, L, T, V
Lee^26^(FASEB)	2006	OVA induced BALB/c mice	BALF, blood (I)	6	6	IC87114	0.1, 1 mg/kg	E, N, L, M, T, 4, 5, 13, I, α, Eo, 1β
Park^27^	2010	OVA induced C57BL/6 mice,	BALF	7	7	IC87114	0.1, 1 mg/kg	E, N, L, T

Abbreviations: Exp: experimental; Con: control; BALF: bronchoalveolar lavage fluid; OVA: ovalbumin; BMMC: bone marrow mononuclear cells; HUVEC: human umbilical vein endothelial cells; E: eosinophils; N: neutrophils; L: lymphocytes; M: macrophages; T: total cell counts; I: IgE; α: TNF-α; 2: IL-2; 4: IL-4; 5: IL-5; 6: IL-6; 10: IL-10; 12: IL-12; 13: IL-13; γ: IFN-γ; β: TGF-β; 1β: IL-1β; Eo: eotaxin; V, VEGF.

**Figure 1:**
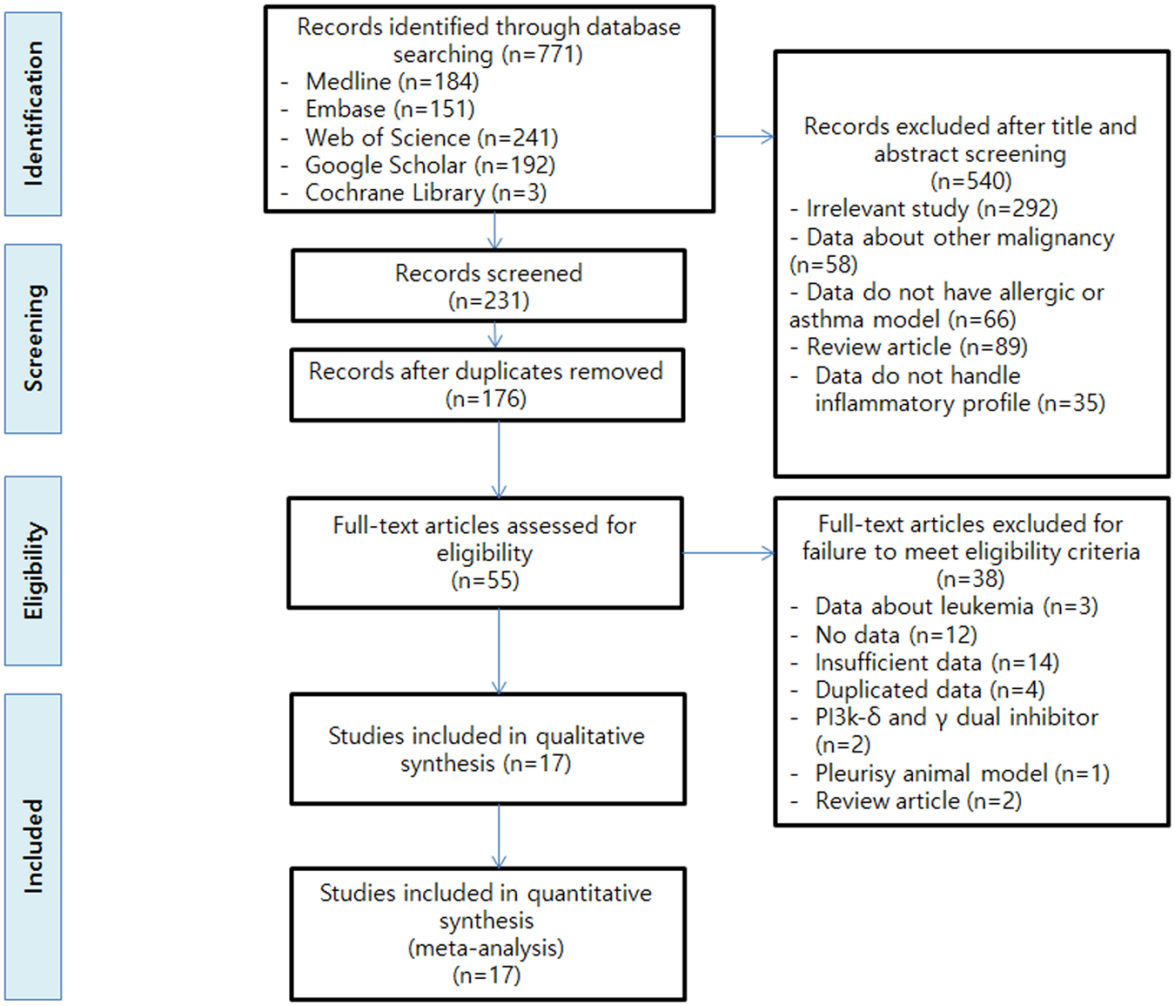
Flow chart for the study selection process according to Preferred Reporting Items for Systematic Reviews and Meta-Analyses (PRISMA) guidelines.

Eleven of these studies were on PI3K pan-inhibitors (Table 1), and six were on PI3K-δ inhibitors (Table 2). Primary outcomes were cell counts and inflammatory profile. We evaluated the effects of PI3K pan-inhibitors and PI3K-δ inhibitors as subgroups. The PRISMA diagram with the main selection process and reasons for exclusion is shown in Fig. 1.

### Effector cell counts

#### Total cell counts

**Figure 2:**
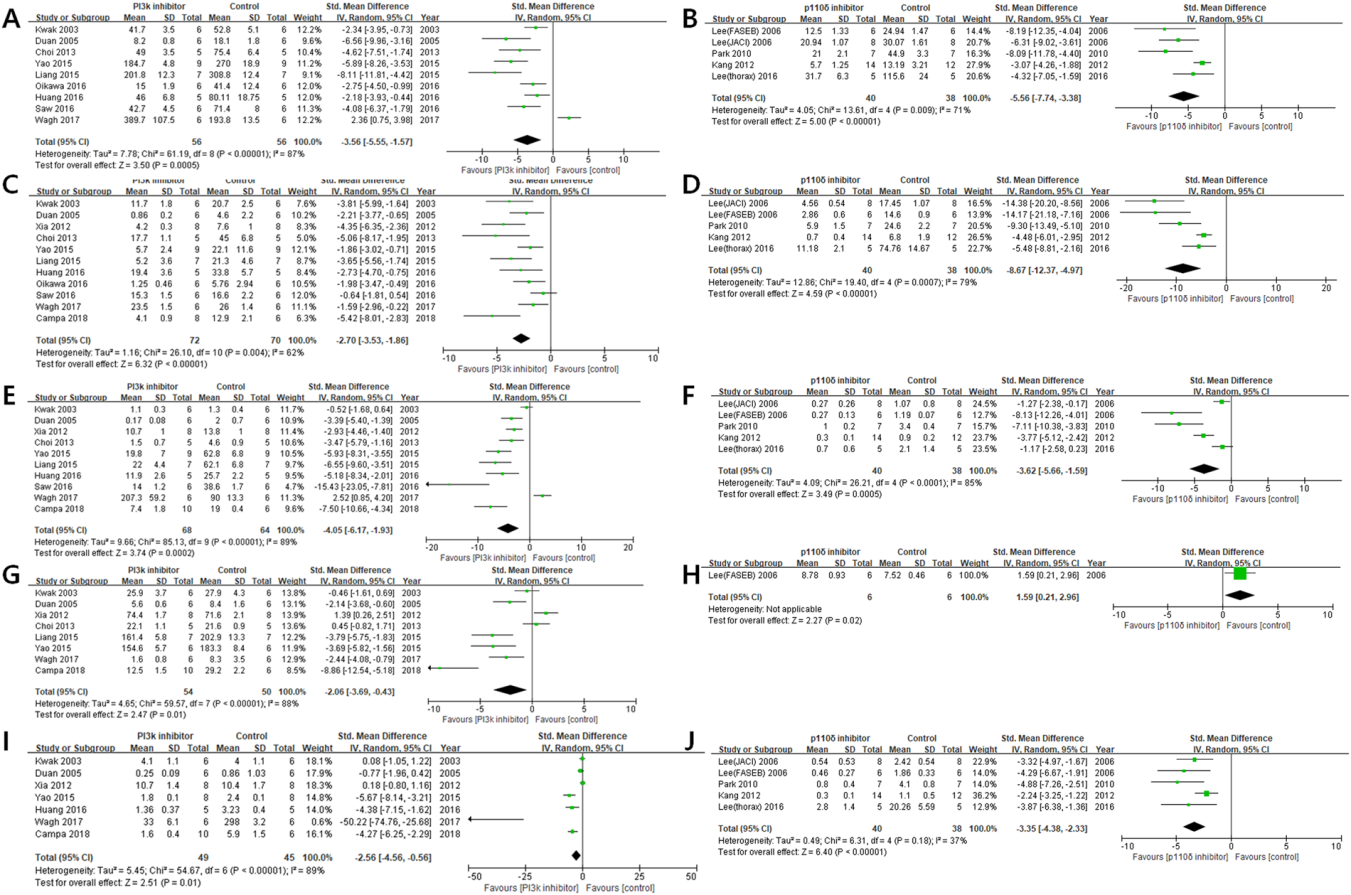
Forest plots regarding effector cell counts showing the effect of: (**A**) PI3K pan-inhibitors on total cell counts; (**B**) PI3K-δ inhibitors on total cell counts; (**C**) PI3K pan-inhibitors on eosinophil counts; (**D**) PI3K-δ inhibitors on eosinophil counts; (**E**) PI3K pan-inhibitors on neutrophil counts; (**F**) PI3K-δ inhibitors on neutrophil counts; (**G**) PI3K pan-inhibitors on macrophage counts; (**H**) PI3K-δ inhibitors on macrophage counts; (**I**) PI3K pan-inhibitors on lymphocyte counts; (**J**) PI3K-δ inhibitors on lymphocyte counts.

The efficacy of the PI3K pan-inhibitors on total cell counts was SMD: −3.56 [95% CI: −5.55 to −1.57] with substantial heterogeneity (I^2^ = 87%) in a total of 9 articles (Fig. 2A). As a subgroup analysis, the efficacy of the PI3K-δ inhibitors on total cell counts was SMD: −5.56 [95% CI: −7.74 to −3.38] with moderate heterogeneity (I^2^ = 71%) in a total of 5 articles (Fig. 2B).

#### Eosinophils

The efficacy of the PI3K pan-inhibitors on eosinophil counts was SMD: −2.70 [95% CI: −3.53 to −1.86] with moderate heterogeneity (I^2^ = 62%) in a total of 11 articles (Fig. 2C). As a subgroup analysis, the efficacy of the PI3K-δ inhibitors on eosinophil counts was SMD: −8.67 [95% CI: −12.37 to −4.97] with substantial heterogeneity (I^2^ = 79%) in a total of 5 articles (Fig. 2D).

#### Neutrophils

The efficacy of the PI3K pan-inhibitors on neutrophil counts was SMD: −4.05 [95% CI: −6.17 to −1.93] with substantial heterogeneity (I^2^ = 89%) in a total of 10 articles (Fig. 2E). As a subgroup analysis, the efficacy of the PI3K-δ inhibitors on neutrophil counts was SMD: −3.62 [95% CI: −5.66 to −1.59] with substantial heterogeneity (I^2^ = 85%) in a total of 5 articles (Fig. 2F).

#### Macrophages

The efficacy of the PI3K pan-inhibitors on macrophage counts was SMD: −2.06 [95% CI: −3.69 to −0.43] with substantial heterogeneity (I^2^ = 88%) in a total of 8 articles (Fig. 2G). As a subgroup analysis, the efficacy of the PI3K-δ inhibitors on macrophage counts was SMD: 1.59 [95% CI: 0.21 to 2.96] in a total of 1 article (Fig. 2H).

#### Lymphocytes

The efficacy of the PI3K pan-inhibitors on lymphocyte counts was SMD: −2.56 [95% CI: −4.56 to −0.56] with substantial heterogeneity (I^2^ = 89%) in a total of 7 articles (Fig. 2I). As a subgroup analysis, the efficacy of the PI3K-δ inhibitors on lymphocyte counts was SMD: −3.35 [95% CI: −4.38 to −2.33] with low heterogeneity (I^2^ = 37%) in a total of 5 articles (Fig. 2J).

### Inflammatory cytokines and chemokines

#### Total IgE

**Figure 3:**
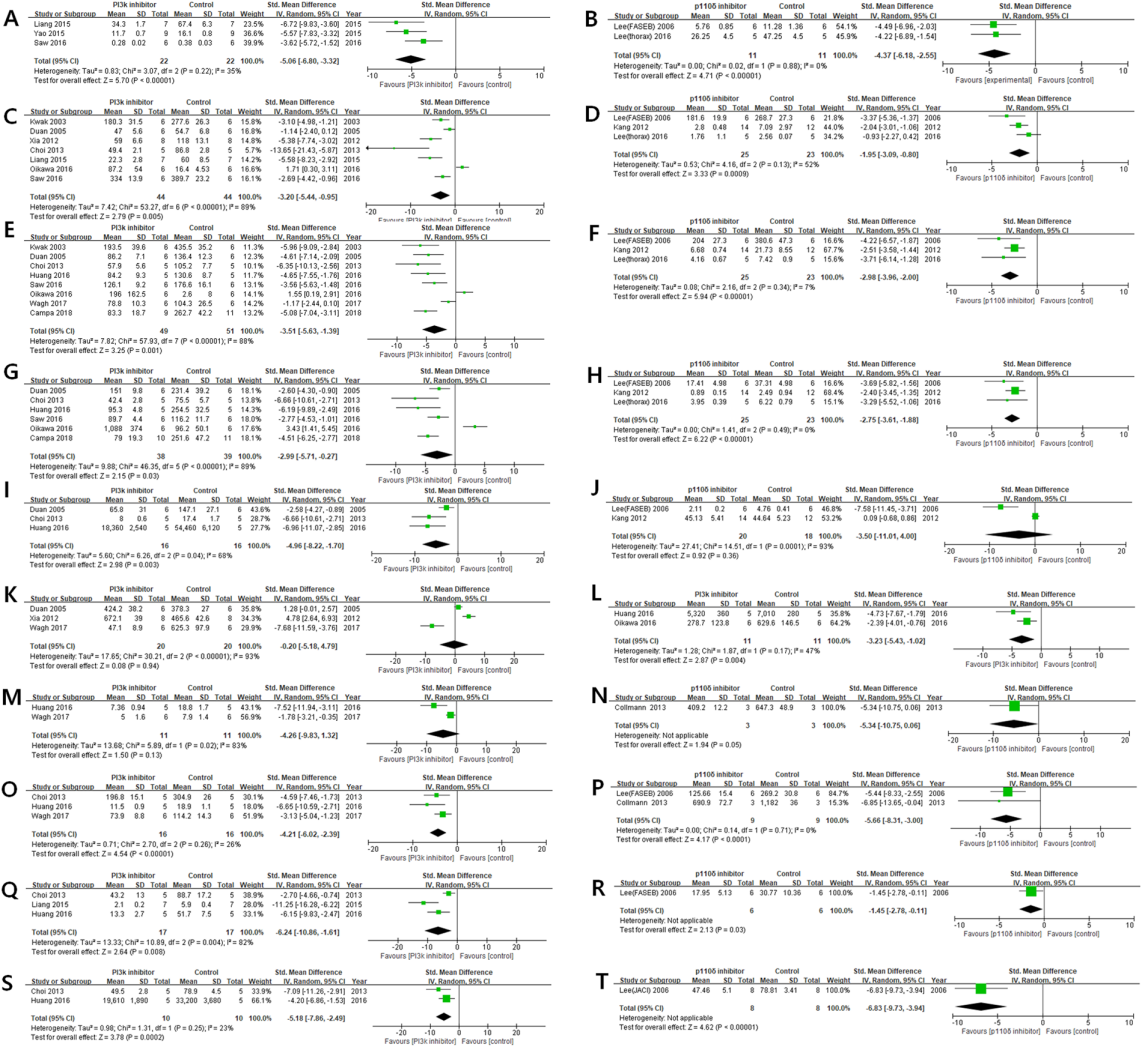
Forest plots regarding inflammatory cytokines and chemokines showing the effect of: (**A**) PI3K pan-inhibitors on total IgE; (**B**) PI3K-δ inhibitors on total IgE; (**C**) PI3K pan-inhibitors on IL-4; (**D**) PI3K-δ inhibitors on IL-4; (**E**) PI3K pan-inhibitors on IL-5; (**F**) PI3K-δ inhibitors on IL-5; (**G**) PI3K pan-inhibitors on IL-13; (**H**) PI3K-δ inhibitors on IL-13; (**I**) PI3K pan-inhibitors on eotaxin; (**J)** PI3K-δ inhibitors on eotaxin; (**K**) PI3K pan-inhibitors on IFN-γ; (**L**) PI3K pan-inhibitors on TGF-β; (**M**) PI3K pan-inhibitors on IL-6; (**N**) PI3K-δ inhibitors on IL-6; (**O**) PI3K pan-inhibitors on TNF-α; (**P**) PI3K-δ inhibitors on TNF-α; **(Q**) PI3K pan-inhibitors on IL-1β; (**R**) PI3K-δ inhibitors on IL-1β; (**S**) PI3K pan-inhibitors on VEGF; (**T**) PI3K-δ inhibitors on VEGF.

Among the inflammatory profiles, the efficacy of the PI3K pan-inhibitors on total IgE was SMD: −5.06 [95% CI: −6.80 to −3.32] with moderate heterogeneity (I^2^ = 35%) in a total of 3 articles (Fig. 3A). As a subgroup analysis, the efficacy of the PI3K-δ inhibitors on total IgE was SMD: −4.37 [95% CI: −6.18 to −2.55] with no heterogeneity (I^2^ = 0%) in a total of 2 articles (Fig. 3B).

#### IL-4

Among the inflammatory profiles, the efficacy of the PI3K pan-inhibitors on IL-4 was SMD: −3.20 [95% CI: −5.44 to −0.95] with substantial heterogeneity (I^2^ = 89%) in a total of 7 articles (Fig. 3C). As a subgroup analysis, the efficacy of the PI3K-δ inhibitors on IL-4 was SMD: −1.95 [95% CI: −3.09 to −0.80] with moderate heterogeneity (I^2^ = 52%) in a total of 3 articles (Fig. 3D).

#### IL-5

Among the inflammatory profiles, the efficacy of the PI3K pan-inhibitors on IL-5 was SMD: −3.51 [95% CI: −5.63 to −1.39] with substantial heterogeneity (I^2^ = 88%) in a total of 8 articles (Fig. 3E). As a subgroup analysis, the efficacy of the PI3K-δ inhibitors on IL-5 was SMD: −2.98 [95% CI: −3.96 to −2.00] with low heterogeneity (I^2^ = 7%) in a total of 3 articles (Fig. 3F).

#### IL-13

Among the inflammatory profiles, the efficacy of the PI3K pan-inhibitors on IL-13 was SMD: −2.99 [95% CI: −5.71 to −0.27] with substantial heterogeneity (I^2^ = 89%) in a total of 6 articles (Fig. 3G). As a subgroup analysis, the efficacy of the PI3K-δ inhibitors on IL-13 was SMD: −2.75 [95% CI: −3.61 to −1.88] with no heterogeneity (I^2^ = 0%) in a total of 3 articles (Fig. 3H).

#### Eotaxin

Among the inflammatory profiles, the efficacy of the PI3K pan-inhibitors on eotaxin was SMD: −4.96 [95% CI: −8.22 to −1.70] with moderate heterogeneity (I^2^ = 68%) in a total of 3 articles (Fig. 3I). As a subgroup analysis, the efficacy of the PI3K-δ inhibitors on eotaxin was SMD: −3.50 [95% CI: −11.01 to 4.00] with substantial heterogeneity (I^2^ = 93%) in a total of 2 articles (Fig. 3J).

#### IFN-γ

Among the inflammatory profiles, the efficacy of the PI3K pan-inhibitors on IFN-γ was SMD: −0.20 [95% CI: −5.18 to 4.79] with substantial heterogeneity (I^2^ = 93%) in a total of 3 articles (Fig. 3K).

#### TGF-β

Among the inflammatory profiles, the efficacy of the PI3K pan-inhibitors on TGF-β was SMD: −3.23 [95% CI: −5.43 to −1.02] with moderate heterogeneity (I^2^ = 47%) in a total of 2 articles (Fig. 3L).

#### IL-6

Among the inflammatory profiles, the efficacy of the PI3K pan-inhibitors on IL-6 was SMD: −4.26 [95% CI: −9.83 to 1.32] with substantial heterogeneity (I^2^ = 83%) in a total of 2 articles (Fig. 3M). As a subgroup analysis, the efficacy of the PI3K-δ inhibitors on IL-6 was SMD: −5.34 [95% CI: −10.75 to 0.06] in one article (Fig. 3N).

#### TNF-α

Among the inflammatory profiles, the efficacy of the PI3K pan-inhibitors on TNF-α was SMD: −4.21 [95% CI: −6.02 to −2.39] with low heterogeneity (I^2^ = 26%) in a total of 3 articles (Fig. 3O). As a subgroup analysis, the efficacy of the PI3K-δ inhibitors on TNF-α was SMD: −5.66 [95% CI: −8.31 to −3.00] with no heterogeneity (I^2^ = 0%) in a total of 2 articles (Fig. 3P).

#### IL-1β

Among the inflammatory profiles, the efficacy of the PI3K pan-inhibitors on IL-1β was SMD: −6.24 [95% CI: −10.86 to −1.61] with substantial heterogeneity (I^2^ = 82%) in a total of 3 articles (Fig. 3Q). As a subgroup analysis, the efficacy of the PI3K-δ inhibitors on IL-1β was SMD: −1.45 [95% CI: −2.78 to −0.11] in one article (Fig. 3R).

#### VEGF

Among the inflammatory profiles, the efficacy of the PI3K pan-inhibitors on VEGF was SMD: −5.18 [95% CI: −7.86 to −2.49] with low heterogeneity (I^2^ = 23%) in a total of 2 articles (Fig. 3S). As a subgroup analysis, the efficacy of the PI3K-δ inhibitors on VEGF was SMD: −6.83 [95% CI: −9.73 to −3.94] in one article (Fig. 3T).

### Summary

Regarding the cell counts, both the PI3K pan-inhibitors and PI3K-δ inhibitors effectively reduced the total cell counts, eosinophils, neutrophils, and lymphocytes. In contrast to PI3K-δ inhibitors, PI3K pan-inhibitors effectively reduced macrophages.

Regarding the inflammatory cytokines, PI3K pan-inhibitors and PI3K-δ inhibitors effectively reduced total IgE, IL-4, IL-5, IL-13, TNF-α, IL-1β, and VEGF, and had no effect on IL-6. PI3K pan-inhibitors reduced TGF-β and eotaxin, but had no effect on IFN-γ. PI3K-δ inhibitors had no effect on eotaxin.

## Discussion

Before the 1990s, narrative reviews took the role of combining data from multiple studies. A narrative review is a form in which an expert in one field draws conclusions by synthesizing previous studies on a subject. This form has inherent problems of subjectivity due to the lack of transparency. To solve these problems, since the 1990s, there has been a move to apply a new form of systematic review and meta-analysis in various disciplines such as medicine, pharmacy, education, psychology, sociology and ecology^9^. Unlike the narrative review, in which a researcher arbitrarily assigns a weight to each individual study, a meta-analysis secures objectivity by weighting each individual study according to mathematical criteria specially designed in advance. The statistical analysis technique used in the meta-analysis is a transparent, objective, and repeatable framework. This meta-analysis is used to support evidence-based policy and to obtain evidence on the effectiveness of any intervention for a variety of reasons.

In the current study, we present the results of a meta-analytic method which was used to investigate therapeutic effects of PI3K pan-inhibitors/PI3K-δ inhibitors in various experimental murine models of allergic lung inflammation.

Allergic inflammation is orchestrated by complex interplays between diverse cellular components of innate and adaptive immunity in the background of immense pro-inflammatory milieus, in which a wide array of cytokines and chemokines are closely implicated. Bronchial asthma is the hallmark of the allergic inflammatory disorder in the lower respiratory tract^28^. Unfortunately, there is no curable agent currently available for the treatment of allergy and asthma, although numerous therapeutic options have been developed. Some of them are not sufficiently effective for the refractory disease (*i.e*. corticosteroid resistance in patients having severe asthma) and others are efficacious for specific subset of severe asthmatic patients, however, they are high-priced that precludes widespread use of those agents (*e.g.* biologicals that target type 2 cytokines such as IL-4, 5, 13)^29,30^. In this regard, class I PI3Ks have been gaining much attention as promising therapeutic target for allergic disorders because their widespread involvement in controlling nearly all aspects of cellular events, including growth, proliferation, metabolism, motility, and survival^2,4^. Initial studies on the PI3K pathway were mainly driven by cancer biologists^31^. Furthermore, with the increasing knowledge on this pathway, PI3K-targeted therapies using PI3K pan-inhibitor have revealed that the PI3K pathway are closely implicated in a broad spectrum of immune/inflammatory diseases including allergy and bronchial asthma^21^.

Meanwhile, crucial involvement of certain isoforms of PI3Ks in normal physiologic process (*e.g*. genetic knockdown of PI3K-α and -β isoforms leads to embryonic lethality^32,33^) raised concerns on the use of PI3K pan-inhibitors for therapeutic purpose due to its non-selectivity enough to cause systemic adverse effects. In this context, therapeutic blockade of specific isoform of PI3Ks such as PI3K-δ has been intensively studied particularly in allergic inflammation^26,34^, given its preferential expression in hematogenous immune/inflammatory cells such as leukocytes. This approach may reduce potential harmful effects mediated through interfering the normal physiologic and protective inflammatory responses against invading microorganisms. Nonetheless, limited information exists regarding comparative analysis on the therapeutic effects of PI3K pan-inhibition and δ isoform selective inhibition in the treatment of allergic lung inflammation, partly because many of these agents are in the early stage of development, so that their clinical efficacies in real practice are not characterized thoroughly yet^2,5^. Thus, through evaluating their therapeutic effects on multifaceted process of allergic inflammation in pre-clinical experimental models, we can get much information on their comparative efficacies, and thus this may facilitate the development of a novel PI3K-targeted therapy. Particularly, considering the cost and time of each animal experiment, we were interested in new research methods that could improve the integration of results from previous studies. In this regard, a systematic review and meta-analysis of PI3K pan-inhibitors and PI3K-δ inhibitors in animal studies was thought to be an attractive and novel beneficial approach.

As for diverse cellular components of allergic lung inflammation, our results showed that PI3K pan-inhibitors and PI3K-δ inhibitors effectively reduced total cell counts, neutrophils, lymphocytes, eosinophils, and macrophages. Eosinophils are leukocytes that have multiple functions in the host defence and are also involved in immune regulation. Neutrophils are also major key factors in the epithelial barrier in allergic disease and are associated with the severity of allergic asthma^35,36^. Eosinophils are also involved in the production of inflammatory mediators while releasing toxic granule proteins such as eosinophil cationic protein, eosinophil peroxidase, and eosinophil-derived neurotoxin^37^. The emergence of eosinophils from the bone marrow is a particularly important process in the allergic inflammatory reaction, and is regulated by IL-5^14^. PI3K pan-inhibitors are known to inhibit this process. Although the PI3K pan-inhibitors have been shown to effectively lower neutrophils and eosinophils, it has been shown that PI3K-δ selective inhibitors also effectively lower both effector cells.

In particular, IL-4, -5 and -13 are produced in Th2 cells and are associated with allergic hyperresponsiveness and are deeply involved in airway inflammation through eosinophil activation^38^. IL-4, -5, and -13 are known to play crucial roles in the production, migration, survival, and activation of eosinophils. Our results have shown that both PI3K pan-inhibitors and PI3K-δ selective inhibitors effectively lowered IL-4, IL-5, and IL-13, and also effectively lowered the number of eosinophils, the major effector cell.

Classically, IL-1β and TNF-α are necessary for the enhancement of eosinophil migration, activation, and survival, and are necessary for the enhancement of allergic hyperresponsiveness^18^. In fact, IL-1β has been shown to be involved in inflammatory diseases. The inflammasome promotes the maturation and secretion of the pro-inflammatory cytokine IL-1β. TNF-α has similar biologic activity, and both are involved in innate immunity^39–41^. Our study has shown that PI3K pan-inhibitors effectively inhibited both IL-1β and TNF-α, and PI3K-δ selective inhibitors can also inhibit these two important cytokines.

VEGF is a vascular endothelial growth factor that is involved in the formation of blood vessels around the bronchus, and is involved in airway oedema and narrowing, and eventually participates in airway vascular remodelling^42^. In patients with acute asthma, it has been found that VEGF is elevated^43^. In addition, the level of VEGF in asthma patients is highly correlated with disease activity and has been found to be inversely proportional to the diameter of the airway^44^. In our study, both PI3K pan-inhibitors and PI3K-δ inhibitors effectively lowered VEGF.

TGF-β is involved in airway remodelling and fibrosis, and is involved in direct smooth muscle contraction and obstruction of the airways^45^. TGF-β is known to be an important factor involved in tissue fibrosis in asthma^14^. PI3K pan-inhibitors effectively lowered TGF-β, however, there was no data with regard to PI3K-δ inhibitors.

IL-6 is a multifunctional cytokine whose function is to regulate immune response, hematopoiesis, and inflammation^39^. It plays an important role in differentiation of naive T cells into Th17 lymphocytes; however, both PI3K pan-inhibitors and PI3K-δ inhibitors had no effect on IL-6.

In conclusion, selective PI3K-δ inhibitors have at least the same or better efficacy compared with PI3K pan-inhibitors against effector cells and inflammatory mediators. Since the single pathway inhibitors (PI3K-δ inhibitors) can be as effective as PI3K pan-inhibitors, it is recommended to apply them to allergies and asthma. Importantly, we have found that most of the major inflammatory actions associated with PI3K signalling, known as the critical pathway, are mediated by the delta isotype pathway. Our results will be helpful in future clinical trials studying the PI3K-δ signalling pathway.
